# Effects of urethane and isoflurane on the sensory evoked response and local blood flow in the early postnatal rat somatosensory cortex

**DOI:** 10.1038/s41598-021-88461-8

**Published:** 2021-05-05

**Authors:** Viktoria Shumkova, Violetta Sitdikova, Ildar Rechapov, Alexey Leukhin, Marat Minlebaev

**Affiliations:** 1 Laboratory of Neurobiology, Kazan Federal University, Kazan, 420000 Russia; 2 OpenLab of Neurobiology, Institute of Fundamental Medicine and Biology, Kazan Federal University, Kazan, 420000 Russia; 3 Laboratory of Neuromorphic computing and neurosimulations, Institute of Information Technology and Intelligent Systems, Kazan Federal University, Kazan, 420000 Russia; 4 INMED, INSERM, Aix-Marseille University, Marseille, 13273 France

**Keywords:** Biological techniques, Neuroscience

## Abstract

Functional studies in the central nervous system are often conducted using anesthesia. While the dose-dependent effects of anesthesia on neuronal activity have been extensively characterized in adults, little is known about the effects of anesthesia on cortical activity and cerebral blood flow in the immature central nervous system. Substitution of electrophysiological recordings with the less-invasive technique of optical intrinsic signal imaging (OIS) in vivo allowed simultaneous recordings of sensory-evoked functional response and local blood flow changes in the neonatal rat barrel cortex. Using OIS we characterize the effects of two widely used anesthetics—urethane and isoflurane. We found that both anesthetics suppressed the sensory-evoked optical intrinsic signal in a dose-dependent manner. Dependence of the cortical response suppression matched the exponential decay model. At experimental levels of anesthesia, urethane affected the evoked cortical response less than isoflurane, which is in agreement with the results of electrophysiological recordings demonstrated by other authors. Changes in oxygenation and local blood flow also showed negative correlation with both anesthetics. The high similarity in immature patterns of activity recorded in different regions of the developing cortex suggested similar principles of development regardless of the cortical region. Therefore the indicated results should be taken into account during functional explorations in the entire developing cortex. Our results also point to urethane as the anesthetic of choice in non-survival experimental recordings in the developing brain as it produces less prominent impairment of cortical neuronal activity in neonatal animals.

## Introduction

Anesthesia suppresses neuronal activity in the brain structures and impairs their communication, this is associated with changes in the local cerebral blood flow (CBF)^1,2^. The activity of some functional networks is preserved under anesthesia, while others are strongly suppressed^3^. By acting through various mechanisms, general anesthetics alter cortical responsiveness to sensory inputs, alter functional connectivity, and produce a loss of consciousness^4–6^. While light anesthesia modulates cortical functioning, deep anesthesia can induce coma and the pathological burst suppression pattern^5,7–11^. The mechanisms of commonly used anesthetics have been extensively studied in the adult brain. Experimental results show that the effect of anesthesia on cortical activity is drug- and dose-dependent^12–14^. Functional connectivity studies using different anesthesia protocols showed they modulate connectivity patterns in unique ways^6^. Compared to the adult brain, the developing brain is more prone to anesthetic complications, because of the less-effective drug-metabolism and narrow therapeutic windows for commonly used anesthetics^15^. Many of the anesthetic agents that showed efficiency in adult rodents (ketamine, pentobarbital, and the fentanyl-droperidol combination) are limited in use in neonatal rats because they often lead to inadequate anesthetic depth and have a high mortality rate^16^. Therefore, in spite of the variety of anesthetic agents that can be used to produce general anesthesia or sedation during experiments, the well-known anaesthetics isoflurane and urethane are still commonly used in exploration of the developing brain in rodents.

In contrast to adults, in neonates the activity of the nervous system is characterized by a high level of discontinuity. Transient immature patterns of activity are separated by long-lasting periods of neuronal silence^17–19^, suggesting that the manifestations of anesthesia on immature cortical activity may be different. Electrophysiological recordings in the visual system showed strong suppression of the light evoked cortical response even at low doses of isoflurane (0.3–1%) in P10-11 rats, however, the response was moderately facilitated in P13 animals^20^, suggesting developmental changes in the effects of anesthesia in the immature nervous system. The suppressing effect of isoflurane on cortical activity patterns was also shown in the barrel cortex during the first two weeks of postnatal development in rats^21^. At a low dose of isoflurane spontaneous activity was weakly modified, while a 1.9–2.3% concentration of isoflurane strongly suppressed immature patterns of cortical activity, supporting the graded effect of anesthesia on cortical neuronal activity in neonatal rats, as in adult rats. Recordings of spontaneous cortical activity in neonatal mice showed that the administration of isoflurane or urethane reduced the active periods of neonatal activity without significant alteration of the spectral features of the cortical activity patterns and neuronal firing rate^22^. Therefore, while the effects of general anesthesia on the functioning of the central nervous system have been thoroughly characterized in adults, the developmental aspects of their action remain less well understood.

Cerebral blood flow is also an object of dose- and drug dependent modulation by sedatives, analgesics, and anesthetics^23–26^. A dose-dependent CBF reduction in the mouse brain was shown for isoflurane^27^. It was also demonstrated that isoflurane anesthesia evoked a higher baseline CBF during stimulation of the cerebral cortex^8^. Urethane shows minimal effects on the cardiovascular and respiratory systems^28^. While in adults anesthesia acts drug- and dose-dependently, little is known about the effects of different anesthetic protocols on the cortical activity and associated CBF changes in the developing central nervous system.

Using optical intrinsic signal (OIS) recordings to characterize the changes in cortical activity and cerebral blood flow with different types of anesthesia, we attempted to answer this question. Single light wavelength OIS is a simple and powerful less invasive technique to study brain activity^29^. In clinical studies, completely non-invasive implementation of OIS recordings (functional Near-Infrared Spectrosopy, fNIRS) is a routine technique for brain studies^30,31^. Those techniques mainly reflect the hemodynamic cortical response and metabolism, however modulation of light scattering associated with the neuronal activity also contributes to the generation of the intrinsic signal^32^. In studies in adults, conventional OIS recordings and fNIRS are considered to be effective substitutes for the blood oxygenation level-dependent (BOLD) signal recorded by functional magnetic resonance imaging (fMRI)^33,34^.

Decomposition of multispectral OIS recordings allows the extraction of both the hemodynamic and light scattering responses^35–38^. The contribution of different chromophores (oxy- and deoxyhemoglobins), and tissue light scattering (LS) suggested various mechanisms underlying the signal generation recorded by OIS and BOLD fMRI. This may explain the efficiency of the OIS approach in neonatal rats, compared to BOLD fMRI^39–41^. OIS imaging carries rich information about neuronal activity, which manifests from several processes that dictate light propagation through biological tissue (absorption and scattering). While the light absorption signal reflects the changes in blood oxygenation related to neuronal activity, LS is linked to changes in the optical properties of neural tissue as a secondary consequence of neural excitation. In the mature brain, the LS response is overlapped in time and in amplitude by the hemodynamic response. Because of the immaturity of the nervous and vascular systems, particularly the immaturity of neurovascular coupling, both neural tissue and hemodynamic responses are much slower and separated in time^37,42^. The early phase of OIS, during the first seconds of the cortical response, is characterized by light scattering changes^40^. The high correlation of the optically recorded signal with the power of the evoked neuronal oscillatory response in the immature barrel cortex strongly supports the idea that in the developing brain OIS imaging may serve as an approach for direct measurement of cortical functional response. The second phase of the OIS response, characterized by changes in oxy- and deoxyhemoglobins, indicates changes in CBF^43^. Therefore, in the developing nervous system OIS can be used for characterization of the changes in evoked cortical activity and cerebral blood flow.

Therefore, from the experimental point of view, it is critically important to know own effect of drugs on cortical functioning and local hemodynamics for correct interpretation of the results. In the present study, we demonstrate the effects of isoflurane and urethane on sensory evoked functional response and the associated local blood flow changes. We report that in the immature somatosensory cortex both isoflurane and urethane induce dose- and drug dependent alterations in sensory evoked functional response and the associated local blood flow changes.

## Results

### OIS dependence on anesthesia concentration in neonatal rat pups

**Figure 1:**
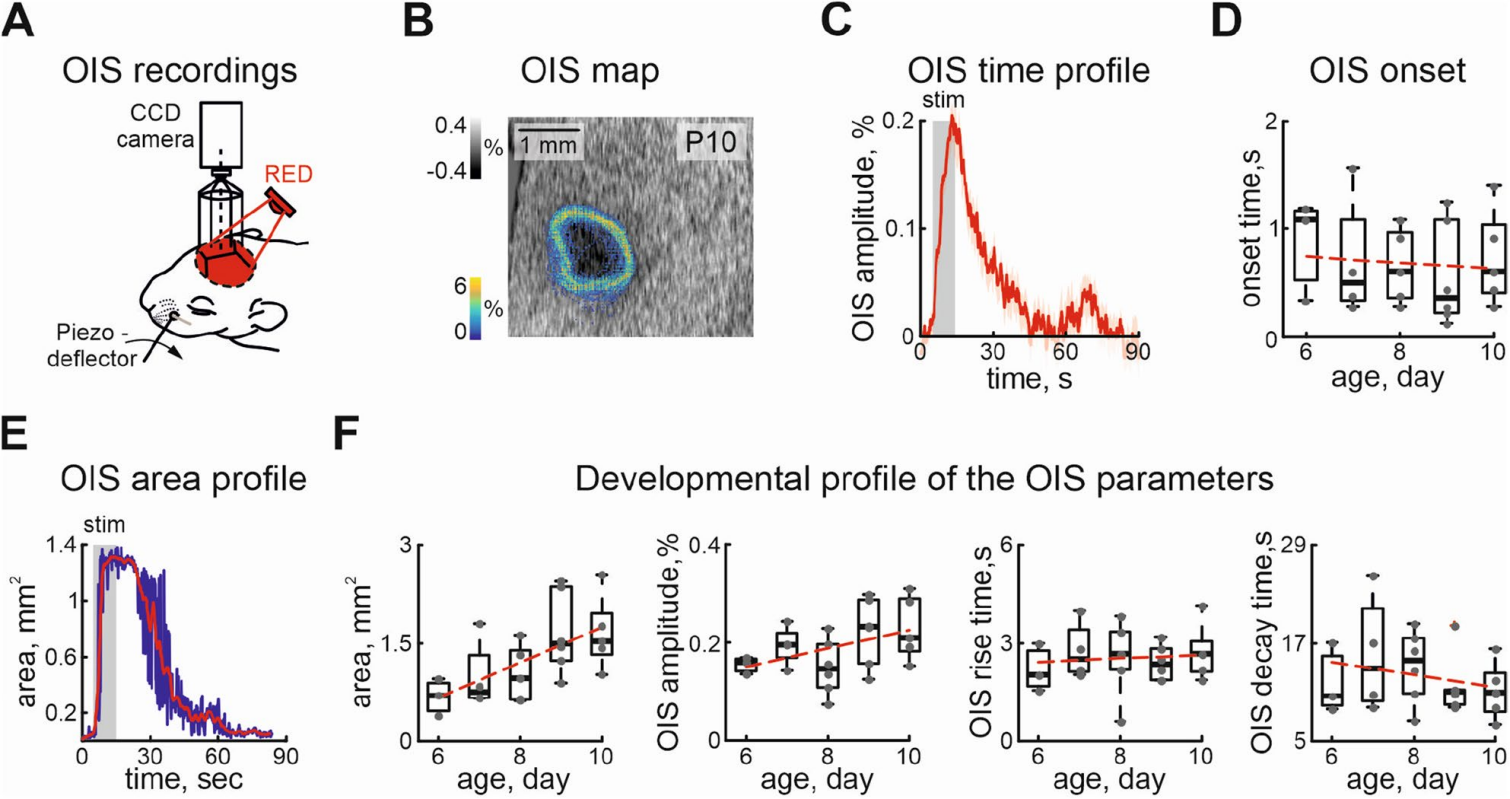
Evoked optical intrinsic signal in the somatosensory cortex of the neonatal rat. (**A**) Diagram of the experimental setup for recording evoked OIS in the barrel cortex of the neonatal rat, (**B**) color-coded density map of the detected OIS contours overlaid on the recorded OIS, seen as a dark spot, (**C**) example of the temporal profile of the OIS recorded using red light, stimulation period is shown by the grey rectangle, mean OIS dynamic is shown by the red line, the shaded area corresponds to the confidence interval, (**D**) developmental changes in OIS onset (0 is the start of stimulation), (**E**) example of OIS area changes during recording is shown by the blue line, the red line is a result of convolution with a Kaiser window of 12 points size, (**F**) developmental changes in the evoked OIS area, amplitude, rise and decay times. The red dashed line is a linear fit. Grey dots are results from individual experiments. Box plots show median (bold black line), the bottom and top edges of the box indicate the 25th and 75th percentiles, The whiskers extend to the most extreme data points not considered outliers, and the outliers are plotted individually using the ’+’ symbol.

To characterize the amplitude-temporal parameters of the OIS evoked in the barrel cortex of neonatal rat pups, conventional OIS recording with red light illumination was used^29,44^ (Fig. 1A). A train of rhythmic vibrissa deflections reliably evoked a drop in reflected light that spanned over tens of seconds (see Table 1, Fig. 1B,C), in agreement with the previous results^39,40^. To estimate the cortical area involved in the evoked cortical response we developed an approach based on kernel density estimation and detected the contours of the OIS (see “Materials and method” section for details).

**Table 1:** The amplitude-temporal parameters of the OIS.

Parameter	Median		25%		75%		Spearmans coefficient, $\rho$	Significance, $\alpha =0.05$
Age	P6	P10	P6	P10	P6	P10		
OIS duration (s)	60.48	40.49	39.76	34.76	64.28	42.2	$-0.26$	Non significant
OIS onset (s)	1.08	0.6	0.52	0.4	1.16	1.04	$-0.08$	Non significant
OIS amplutide (%)	0.16	0.21	0.14	0.18	0.17	0.29	0.36	Significant
OIS rise time (s)	2.04	2.68	1.68	2.12	2.76	3.04	0.05	Non significant
OIS decay time (s)	10.52	11	9.32	8.56	15.44	13.4	$-0.12$	Non significant

In spite of the variation of the detected contours between the frames, the maximal contour density coincided with the visually detected OIS (Fig. 1B). OIS onset was slightly delayed compared to the start of stimulation (Table 1, Fig. 1C,D). Analysis of the onset times between animals of different ages does not show significant developmental changes (Table 1). The cortical area involved in OIS quickly reached a peak size dependent on the animal’s age. In P6 rat pups the mean OIS area was less than 1 ${\text{ mm }}^2$ ($0.68 \pm 0.17\,{\text{ mm }}^2$), by P10 evoked OIS covered larger cortical territories ($1.65\pm 0.25 \, {\text{ mm }}^2$, $\rho =0.59, p < 0.05$, n = 23 P6-10 rat pups), this is in an agreement with the developmental increase of the barrel cortex (Fig. 1E,F).

Though there is a positive correlation of OIS area and animal maturation, we have not found significant changes in the OIS amplitude during animal development (Table 1, Fig. 1F). No significant differences were found for the rise time of the OIS nor for the decay time (Table 1, Fig. 1F). We grouped recordings in spite of the different ages of the rat pups, due to the lack of the developmental changes in OIS parameters between the animals.

**Figure 2:**
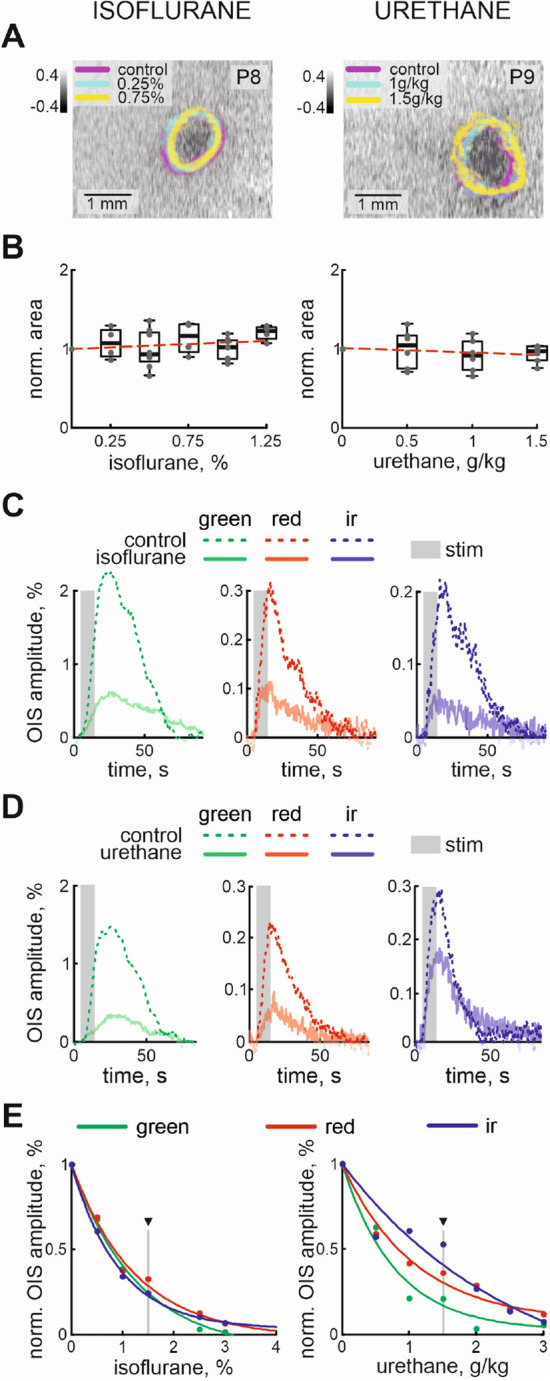
Concentration dependence of OIS on isoflurane and urethane. (**A**) Examples of OIS recorded using red light in control and under 0.25%, 0.75% isoflurane (left) and 1 g/kg, 1.5 g/kg urethane (right) anesthesia. OIS contours in control and under different concentrations of the anesthetics are colour-coded. Group data of normalized red OIS area for changes in concentration of isoflurane and urethane are shown in (**B**). OISs recorded using different light wavelengths (Green, Red and IR) in control and under 1.5% isoflurane (**C**) and 1.5 g/kg urethane (**D**). The stimulation period is shown by the grey rectangle. Light wavelength is colour-coded. OISs recorded in control conditions are shown by dashed line, while OISs traces under anesthesia are shown by semitransparent lines of the corresponding colour. (**E**) Dependencies of the normalized amplitudes of the OISs recorded using Green, Red and IR light with different concentrations of isoflurane (left) and urethane (right) are shown. Triangle marks the concentrations at which the examples of multi spectral OISs are shown on (**C**, **D**).

To examine the dependence of evoked OIS on anesthesia, experiments were done with urethane and isoflurane^17,18,39,45^. We gradually increased the concentrations of the anesthetics and monitored the evoked OIS. Firstly, using Red illumination we characterized the cortical area of the OIS. The area of evoked OIS was weakly modulated by different concentrations of isoflurane or urethane ($\rho =0.21$ and $\rho =-0.2$ for isoflurane and urethane, respectively, n = 14 P7-10 rat pups, Fig. 2A,B). Elucidation of the components underlying the OIS requires multi spectral recordings of the signal, therefore experiments were carried out using three types of illumination (Red, Green, and IR). A progressive reduction in OIS amplitude was observed with increasing concentrations of isoflurane or urethane using all three illumination wavelengths (Fig. 2C,D). The dependence of OIS amplitude on the anesthesia concentration matched best with an exponential model for both anesthetics in all types of illumination (${\text{ r }}^{2}$ for OISs recorded using Green, Red and IR lights were 0.99, 0.99, 0.99 and 0.98, 0.98, 0.98, respectively, n = 6 and n = 8 rat pups of P7-10, Fig. 2E).

Since the OIS is calculated using normalization for its pre-stimulation baseline, changes in recorded light intensity (reflection for Red and Green wavelengths, and transmission for IR light) during and before stimulation may affect the calculation of OIS. In order to test the impact of the baseline changes on calculating the dependence of OIS on the anaesthesia concentration, we also estimated the amplitude of the recorded light intensity as a function of the concentration of the anesthetics. The results showed that recorded light intensity during baseline weakly depended on the concentration and type of anesthetic. We did not see developmental changes in OIS baseline dependence on anesthesia concentration (Table 2). Therefore, modulation of the OIS amplitude is exclusively associated with evoked response changes at different concentrations of urethane or isoflurane.

**Table 2:** Developmental changes of OIS baseline dependence on anesthesia (Spearman’s rank correlation coefficient, $\rho$).

	Green illumination	Red illumination	IR illumination
Urethane	$2 \pm 2 \times 10^{-5}$	$0.5 \pm 1 \times 10^{-5}$	$1.5 \pm 1 \times 10^{-5}$
Isoflurane	$-0.5 \pm 1 \times 10^{-6}$	$-1.3 \pm 0.4 \times 10^{-6}$	$-2 \pm 11\times 10^{-5}$

To characterize the developmental changes of the OIS dependencies on the anesthetics the exponential concentration constants (drop in OIS amplitude for *e* time) were calculated. Our results showed that concentration dependence of OIS varies between the wavelength of light used (the average exponential concentration constants for Green, Red and IR OISs were $1.1 \pm 0.1$%, $1.3 \pm 0.2$%, $1.3 \pm 0.2$% for isoflurane and $1.1 \pm 0.1$ g/kg, $1.5 \pm 0.1$ g/kg and $1.6 \pm 0.1$ g/kg for urethane). As the principal chromophores are characterized by different absorption spectra, the difference in concentration constants for Green, Red and IR light wavelengths suggests that the anesthetics affect different OIS mechanisms.

### Dose-dependent changes of the evoked cortical response

There are several mechanisms underlying OISs, and their contribution to the generation of OIS depends on the maturation of the nervous and vascular systems. While in the mature brain the OIS is predominantly determined by the hemodynamic changes following neuronal activity, in the immature nervous system the early part of the OIS is largely defined by the tissue component^40^.

**Figure 3:**
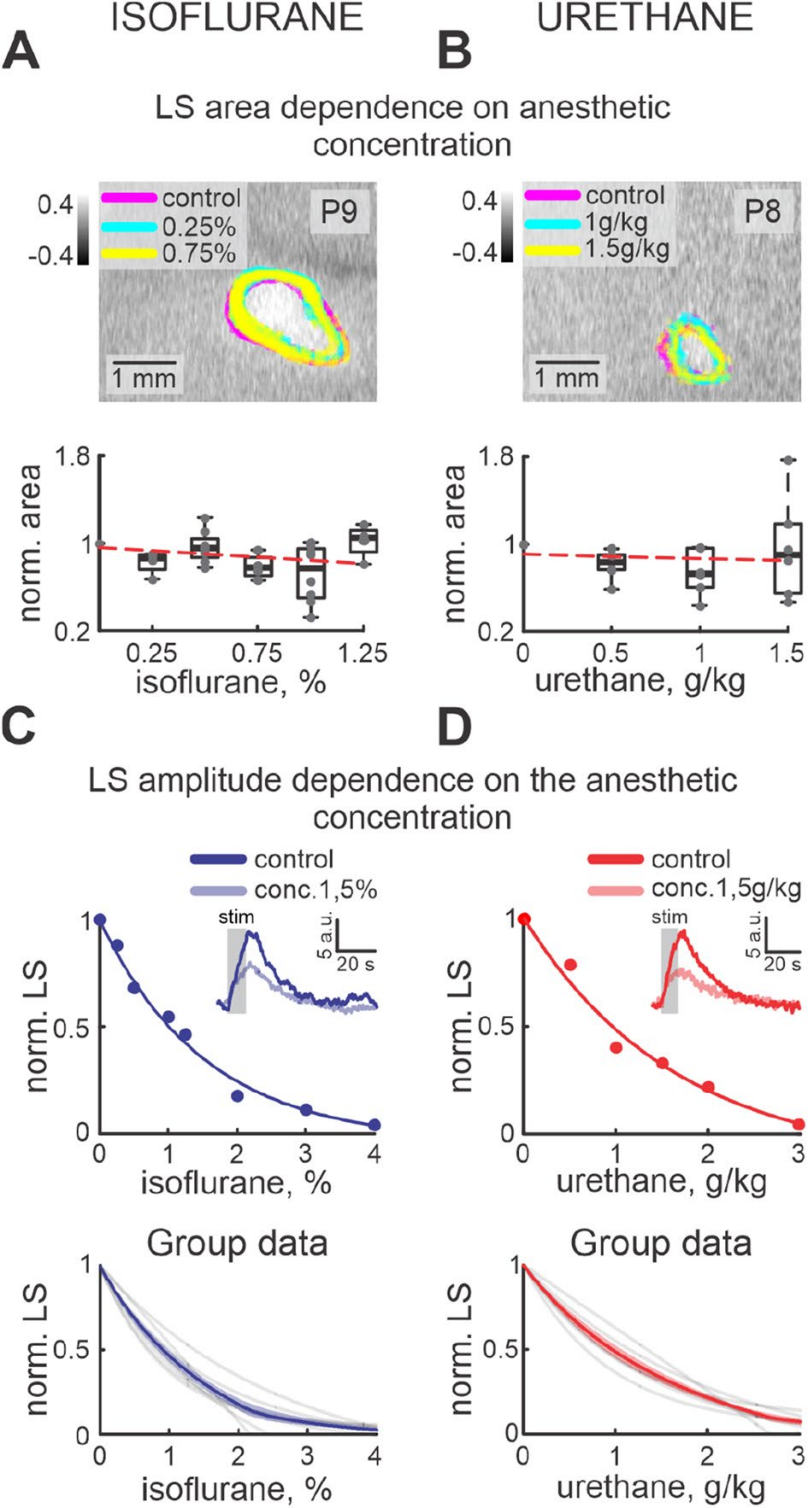
Dose-dependence of functional response on isoflurane and urethane. LS responses in control and under 0.25%, 0.75% of isoflurane (**A**) and 1 g/kg, 1.5 g/kg urethane (**B**) anesthesia. LS responses in control and under different anesthetic concentrations are colour-coded. Group data of normalized area changes are shown by box plot graphs under the examples of LS responses. Individual dependencies of normalized LS changes on different isoflurane (**C**) and urethane (**D**) concentrations are shown in the upper row. Dots correspond to the LS amplitude at different concentrations of anesthetic agent. Exponential model fits are shown by lines of the corresponding colour. Examples of LS changes in time in control and under 1.5% of isoflurane and 1.5 g/kg urethane are shown as insets. Group data are shown in the bottom row. Exponential fits from each experiment are shown by grey lines, while mean fits are shown by blue and red lines for isoflurane and urethane, respectively.

To characterize the effects of urethane and isoflurane on the evoked cortical response in the immature nervous system we estimated the changes in the tissue component (light scattering, LS), local blood flow (total hemoglobin, HbT), and tissue oxygenation level (oxygenated hemoglobin, HbO) in the barrel cortex following sensory stimulation. Analysis of the multi spectral OIS recordings showed that an increase in the concentration of the anesthetics resulted in a reduction of the amplitude of the LS without changes in the cortical area involved in the cortical functional response (Fig. 3A,B). While in control conditions the mean area of the functional response was slightly bigger than 1 ${\text {mm}}^2$ (1.37±0.12 ${\text {mm}}^2$, n = 14 P7-10 rats), under different concentrations of isoflurane or urethane, the area of the LS response changed non significantly by only a few percent ($\rho$ = − 0.21 and $\rho$ = − 0.36, − 1.90±7.66% and 3.79 ± 19.37% from control area for 1.5$\%$ isoflurane and for 1.25 g/kg of urethane, respectively, n = 14 P7-10 rats). The exponential fit model highly matched LS amplitude dependence on anesthetic concentrations ($\hbox {r}^{2}$ 0.99 and 0.99 for urethane and isoflurane, respectively) showing non linear correlation of functional cortical response on depth of anesthesia (*e* concentration constant 1.4±0.1 g/kg for urethane and 1.2±0.1% for isoflurane, n = 14 P7-10 rats, Fig. 3C,D).

**Figure 4:**
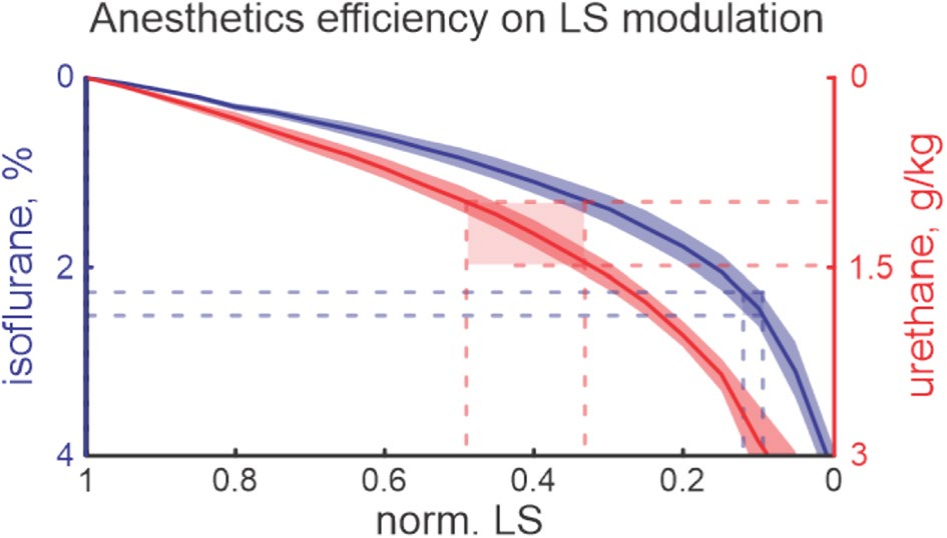
LS modulation by isoflurane and urethane. Functional effects of isoflurane and urethane on LS are shown. The type of anesthetic is colour-coded, the shaded areas correspond to the confidence intervals. Horizontal blue and red lines display the experimental concentration ranges for isoflurane and urethane, respectively. Note, that the experimental concentration of isoflurane suppresses LS by up to seven times, while for the experimental concentration of urethane LS decreased by only 2–3 times.

Though urethane and isoflurane belong to different classes of anesthetic, we compared their efficiency on the evoked functional activity of the barrel cortex Fig. 4. Our results showed that a slight increase in isoflurane concentration in the “subexperimental” concentration range (lower than minimal alveolar concentration, MAC) strongly affected the LS amplitude (an increase of the isoflurane concentration by about $0.23\pm 0.01$% reduced LS by 10 %), while in the MAC range (2.21–2.47%),the isoflurane modulation of LS was less powerful and required an almost threefold increase in concentration to change LS by 10% ($0.83\pm 0.05$%/10% LS, Fig. 4), demonstrating strong changes in isoflurane efficiency at different concentrations. LS dependence on the urethane concentration showed a less pronounced exponential fit. The LS changes in the experimental concentration range (1–1.5 g/kg) and “subexperimental” ranges were smaller ($0.2\pm 0.01$ g/kg per 10% of LS and $0.31\pm 0.01$ g/kg per 10% of LS for “subexperimental” and experimental ranges of urethane concentrations, respectively). We have also found that commonly accepted concentrations for isoflurane and urethane affected LS differently. While urethane at a concentration of 0.9–1.5 g/kg reduced LS by up to three times (to 50–30% from control), an experimental dose of isoflurane reduced the functional cortical response by more than seven times (LS dropped to 13–10% from control) and in some experiments the tissue component was almost completely blocked.

**Figure 5:**
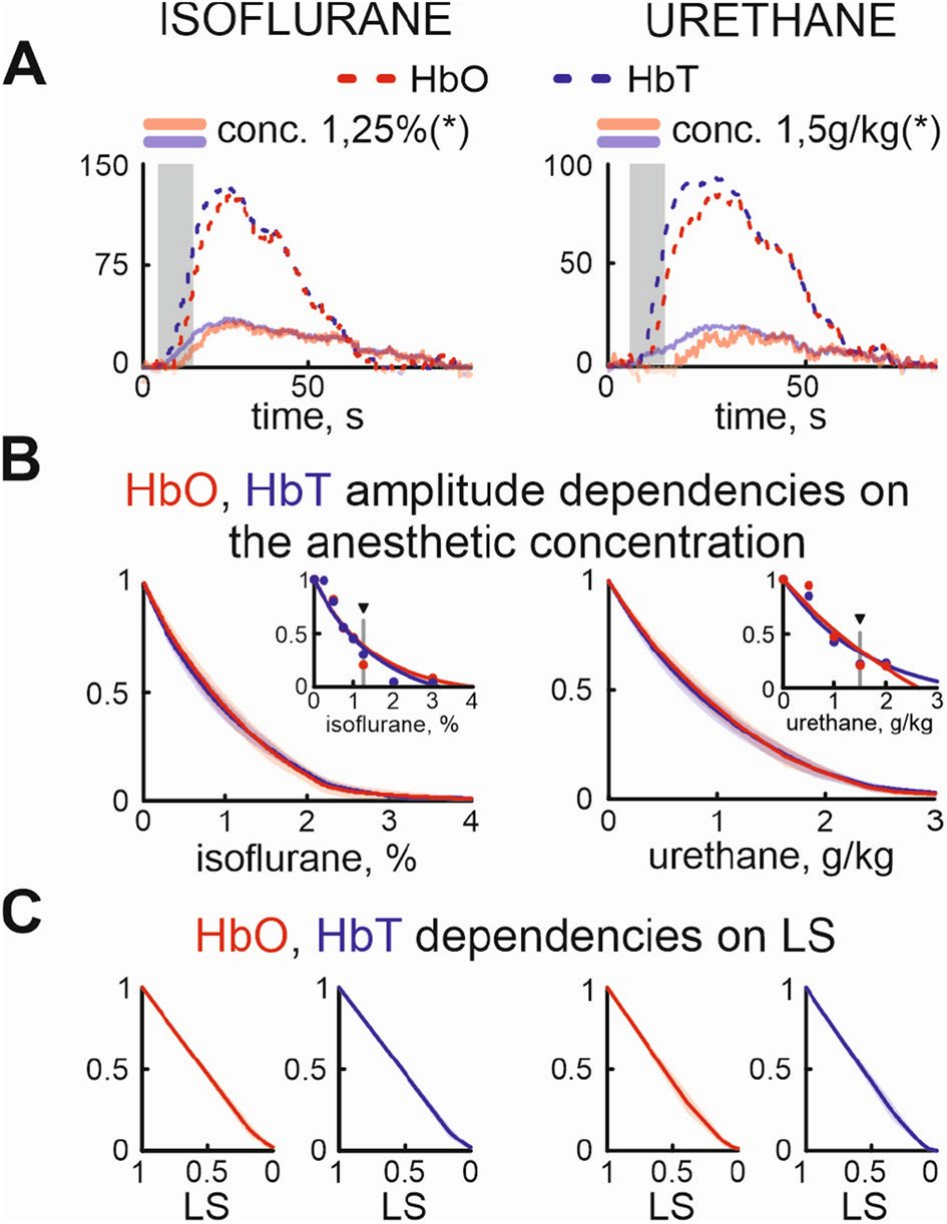
Dependence of local cerebral blood flow on anesthesia concentration and LS changes. (**A**) Oxygenated hemoglobin (HbO, red colour) and total hemoglobin (HbT, blue colour) changes in control and under 1.25% isoflurane (left) and 1.5 g/kg urethane (right). The grey box corresponds to the stimulation period. Control is shown by dashed line of corresponding colour, while semitransparent lines correspond to the anesthesia condition. (**B**) Group data of normalized concentration dependence of HbO, and HbT on isoflurane (left) and urethane (right). Individual examples are shown as insets. Triangles marks the concentrations at which the examples of HbO and HbT are shown on (**A**). (**C**) HbO, HbT dependencies on the LS modified by isoflurane (left pair) or urethane (right pair).

We also analyzed the dose- and anesthetic-dependence of CBF on the different concentrations of urethane and isoflurane. The hemodynamic response following evoked cortical activity also showed negative dose-dependence on both anesthetics (Fig. 5A). Similar to the LS, increase of the anesthesia concentration suppressed local increase of the oxy, and total hemoglobins following whisker stimulation Fig. 5A. Those changes matched the exponential decay model (concentration constant *e* for isoflurane was $1.2 \pm 0.1$%, $1.1 \pm 0.1$% and for urethane $1.1 \pm 0.1$ g/kg, $1.1 \pm 0.1$ g/kg, respectively, Fig. 5B). While a direct effect of anesthesia on cerebral blood flow was shown in adults, we have seen that the hemodynamic response almost linearly correlated with the LS changes (Pearson coefficients for HbO changes under isoflurane $-0.99$ and urethane anesthesia $-0.99$; HbT changes under isoflurane $-0.99$ and urethane $-0.99$, n = 14 p $< 0.01$, Fig. 5C).

## Discussion

The effects of anesthesia on cortical activity and cerebral blood flow in the developing nervous system need to be investigated because of their importance to functional studies in vivo and their interpretation. From this point of view, our results enriched this line of investigation and contributed novel insights into the actions of two widely known anesthetics, urethane and isoflurane. Using the less-invasive technique of intrinsic optical signal imaging in the barrel cortex of the neonatal rat model we show dose-dependent effects of urethane and isoflurane on the cortical evoked response, changes in local blood flow, and oxygenation.

Isoflurane and urethane are anesthetics widely used in neuroimaging and electrophysiological investigations in rodents in vivo, serving to eliminate physiological stress and motion effects. While several studies assessed the effect of anesthesia on immature cortical activity, the fidelity between the different anesthetics and the functional cortical response and cerebral blood flow in neonates have not previously been examined, and therefore warrants thorough investigation and discussion^21,22,46^.

The early postnatal period of somatosensory cortex development is characterized by the expression of unique patterns of immature oscillatory activity. These can be spontaneous, or evoked by stimulation of the sensory receptive fields and observed in the corresponding cortical representations of the body in the somatosensory cortex^17,18,20,47^. These patterns are also seen in the developing visual cortex^20,48^, suggesting that cortical development undergoes similar principles regardless of the cortical region. Based on the high correlation of the OIS with electrophysiological recordings in the developing barrel cortex^40^, we suggest that the observed changes in OIS may reflect the common principles of anesthesia action both on local blood flow and the cortical response, regardless of the cortical region.

We show that both of the anesthetic agents produced a consistent dose-dependent alteration of the cortical evoked response to vibrissa stimulation. This was evidenced by dose-dependent decreases of light scattering of the barrel cortex when receiving multiple sensory stimulation. The anesthetic dose-dependent reduction in evoked and spontaneous activity is shown in previous electrophysiological findings in the adult nervous system^8,14,49,50^.

The cellular and molecular pharmacology of anesthetics has been reviewed extensively, yet the mechanisms underlying the actions of anesthesia are not completely understood. Dose-dependent depression of glutamatergic transmission, and potentiation of GABAa transmission has been shown for both isoflurane and urethane^28,51–55^. Though isoflurane targeting of both excitatory and inhibitory systems is debatable. Isoflurane (as well as other volatile anesthetics such as halothane and sevoflurane) was shown to potentiate GABAa and glycine receptors^56,57^, while AMPA receptors were less affected^58^. However, compared to such intravenous general anesthetics as propofol and etomidate, whose primary target is GABAa receptors, the amplifying effect of isoflurane on GABAa function was less prominent^59,60^. This supports the idea that anesthesia effects produced by isoflurane involves extra mechanisms. Isoflurane was also reported to potently inhibit neuronal nACh receptors^61,62^ and activated two-pore potassium channels, such as TREK-1 (K2P2.1) and TASK (K2P3.1)^63^ that contribute to its anesthetic action. In contrast to isoflurane, urethane showed only a limited effect on nACh receptors^28^. But urethane was shown to depress the intrinsic excitability of layer 4 neurons and increased the shunting potassium background leak conductance^64^. Urethane-dependent current appeared to be through the opening of Ba2+ -sensitive potassium channels, whose properties are consistent with two-pore K+ channels such as TWIK (K2P1.1)^65^. Isoflurane-sensitive TREK-1 and TASK potassium channels did not appear to contribute to the suppressive action of urethane on neuronal activity. Therefore isoflurane and urethane are distinct in their spectrum of action that may explain their differences in anesthesia. In spite of the immaturity of the nervous system and the progressing development of the cortical circuitry, these mechanisms of anesthesia may also play role in the dose dependent reduction of evoked cortical network activity. The exponential model fits observed for both anesthetics display the process of tissue saturation by the anesthesia, which is not agent-specific. A progressive decrease in the apparent saturation level of the LS reflects a progressive suppression of cortical neurons responding simultaneously to sensory stimuli until the neuronal involvement is minimal.

The anesthetic action on thalamic cells (shown during deep anesthesia^66^) which have been hyperpolarized because of the anesthetic action on GABAa or activation of potassium channels^53,67^, may also provide the conditions for the suppression of thalamic input into the barrel cortex. The emergence of the arousal system that occurs around this age also has a direct effect via reduced acetylcholine level^20,68^. Compared to urethane, isoflurane much more efficiently suppressed evoked responses even at low concentrations. While the light scattering component of the OIS indicates neuronal activity, the difference between isoflurane and urethane is likely to result from the unique modulation of cortical response by different anesthetics. Electrophysiological studies showed that immature patterns of evoked activity were weakly modified by urethane in the concentration range of 0.5–1.5 g/kg^39^. But strong suppression of the evoked cortical response by isoflurane was also demonstrated in the developing barrel and visual cortices^20,21^. Interestingly isoflurane suppresses the oscillatory part of the evoked response while the sensory evoked potential was left almost intact. These unique mechanisms of isoflurane action on the sensory evoked potential and the oscillatory part of the response require further investigation. However, inhibition of the late phase of the evoked response may be the result of the combination of suppression of cortical activity and recurrent inhibition from the reticular nucleus to the thalamic principal cells^53^. This had an added effect on the generation of thalamus driven immature oscillatory activity during this developmental period^39^. The less prominent effect of urethane suggests better preservation of the cortical functioning that makes urethane the anesthetic of choice in studies in the developing nervous system. Comparative fMRI studies with different anesthetics also showed that functional connectivity pattern under urethane anesthesia was close to the non-anesthetized animal (only 6% of all connections were significantly suppressed)^6^. Therefore urethane anesthesia exhibits the fewest differences in functional connectivity in the cortex supporting our conclusion of preferential use of urethane.

The previously shown discrepancy of the dose-dependence effects on the evoked cortical activity in mice and rat pups may be due to the maturity of the central nervous system. The advanced maturation of mice compared to the rats explains the difference in the action of anesthesia in these studies^69^. The developmental decline in the efficiency of anesthesia is also supported by the age-dependent decrease in the effect of anaesthesia on the spontaneous and evoked cortical activity in the barrel and visual cortices^20,21^.

In contrast to urethane, which has restricted effect on the cardiovascular system, isoflurane is also known as a vasodilator^70,71^, it is likely that the cerebrovascular reactivity to isoflurane is dose-dependent, even in neonates. However, independently of the type of anesthetic agent used, a linear dependence of the HbO and HbT changes on the LS decrease was observed. Therefore, there were not consistent difference between the effects of isoflurane or urethane on the intensity of the neurally coupled hemodynamic response. There may be developmental changes in the isoflurane provoked vasodilation that are not yet matured at this age in the rat pup. This concept is consistent with studies demonstrating developmental changes in vasomotor tone of the pulmonary artery in the neonatal rat^72^. The suppression of the hemodynamic changes under anesthesia shown are also in agreement with other authors. Early optical imaging experiments revealed that in the awake animal, hemodynamic signals are substantially larger in response to whisker stimulation^73^ or limb stimulation^74,75^ than in anesthetized animals.

Our study revealed that the sensory evoked cortical functional response and local CBF were uniquely modulated by isoflurane and urethane. Our results highlight the necessity of careful monitoring of the anesthesia protocols for reliable comparison between experimental results. We also want to point out that urethane allows better control over the depth of anesthesia, as opposed to isoflurane, and affects the cortical functions in the developing brain less .

## Methods

### Surgery

All animal-use protocols followed the guidelines of Kazan Federal University on the use of laboratory animals (ethical approval by the Institutional Animal Care and Use Committee of Kazan State Medical University N9-2013). Wistar rats of both sexes from postnatal days [P] 6–10 were used (P0 was the day of birth). Surgery was performed under isoflurane anesthesia (5% for induction and 1–2% during surgery). The rat skull was cleaned of skin and periosteum and covered with an acrylic (Meliodent RR) except for a $5 \times {5\,{\text {mm}}^2}$ window above the barrel cortex. A metal ring with an inner diameter of 5 mm was attached to the cement on the head of the animal to make a chamber. Subsequently, the metal ring was fixed into the stereotaxic apparatus. To increase the transparency of the bone in the bottom of the chamber the opened skull part was polished and the chamber was filled with saline and covered with a coverslip. The rats were warmed, surrounded by a cotton nest, heated via a thermal pad (35–${37}^{\circ }{\text {C}}$) and left for an hour to recover from anesthesia.

### Optical intrinsic signal recordings and analysis

OIS was recorded in head-fixed rat pups using a video acquisition system. A CCD camera (QICAM Fast 1394) was positioned above the barrel cortex using stereotaxic coordinates^76^. The camera was focused 400–$800\,{\upmu }{\text{ m }}$ below the skull, which approximately corresponds to the depth of the granular layer of the barrel cortex as indicated by electrophysiological activity^47^. The cortex was illuminated by light emitting diodes (LED) positioned around the microscope objective, managed and synchronized with the camera by an Arduino Uno microcontroller. Video frames were recorded at a resolution of $130 \times 174$ (1 px  = 35 ${\upmu }{\text{ m }}$) and a frame rate of 6 Hz. Single wavelength illumination with a 625 nm LED was used to characterize developmental changes in OIS, while three types of diodes (528 nm, 625 nm and 850 nm herein referred to as Green, Red and IR, respectively) were used to record OIS for analysis of the functional response and cerebral blood flow changes. The actual spectra of the diodes were measured using a Thorlabs CCS175 spectrometer. The effects of anesthesia on the evoked cortical response and CBF were estimated using alternate illumination of the barrel cortex with the three types of diodes (6 Hz/LED). While the Green and Red diodes were positioned above the skull around the head to produce spatially uniform illumination, the IR diode was placed below the head of the animal facing towards the camera. Different wavelengths were used for post-hoc estimation of the volumetric and oximetric changes in hemodynamic response and tissue light scattering changes as previously described^37,40^. OIS was evoked by vibrissa deflections. A train of brief pulses (10 ms pulse duration, 10 s train duration, 90 s inter-train interval, repeated 12–20 times) was applied at a frequency of 2 Hz via a piezo deflector (Noliac). A piece of soft foam on the tip of the piezo bender touched most of the vibrissa on the animal’s contralateral snout, providing simultaneous deflection of multiple whiskers. Data pre-processing included per-frame spatial filtering with a 2D Gaussian filter (sigma = 2 px) and subsequent illumination artifact correction. The illumination correction consisted of, first, calculating the reference time profile averaged over the reference region, followed by its subtraction from each pixel intensity^77^. The pre-processed frames were then averaged across all trials (n = 10–20). The experimental protocol was one-conditioned, so the OIS map was calculated using the first-frame subtraction approach as described previously^29,42^. Preliminary detection of the OIS signal was carried out by an operator based on visual detection of a group of pixels with conjointly changed brightness during the stimulation. The OIS time profile was calculated for the manually chosen OIS center. To facilitate visualization of OIS in reflection mode, the signals were inverted. In the experiments with OIS decomposition for hemodynamic and functional responses, the data were recorded using alternate lighting with three different light wavelengths. To calculate the averaged extinction coefficients of oxy- and deoxyhemoglobin (HbO and HbR), spectrophotometric data on rat blood absorption was used^78^. $\Delta$HbO, $\Delta$HbR, and the tissue component were calculated using the modified Beer-Lambert law as described previously^38,40^.

### Cortical response contour detection

To calculate OIS contours the fragmentation analysis approach was used. Firstly, a threshold level of 95–98% was estimated based on the intensity distribution of all pixels of the entire image, followed by detection of the pixels with intensity exceeding the threshold. Second, kernel density estimation (KDE) was used to estimate the probability density function of these pixels. A group of united pixels with density exceeding half of the probability density function were considered as OIS and the area was calculated. Two additional conditions were used to increase the accuracy of detection: (1) the manually chosen center of OIS should be inside the contour of the automatically detected OIS, (2) the minimal fullness value of the detected contours was also set at 50–90%. Fulfilment of these conditions served as confirmation of the automatically detected OIS. The code for OIS contour detection is provided at the following address (http://github.com/research-team/memristive-spinal-cord/blob/master/analysis/signal_recognition.py). While the amplitude of the recorded OIS was detectable at up to 3 g/kg of urethane or 4% isoflurane, significant detection of OIS contours was possible only at lower concentrations, 1.5 g/kg for urethane or 1.25% for isoflurane, therefore the concentration range of anesthetics for spatial analysis of OIS and its components is limited to 1.5 g/kg of urethane and 1.25% for isoflurane in the text.

### Anesthesia

Two anesthetics (urethane and isoflurane) were used in this study. These anesthetics have different mechanisms of action and are differ in delivery method, both are widely used in neuroscience. During experiments the urethane (Sigma-Aldrich, USA) was delivered via intraperitoneal injection of 15% solution. The volume of injection was calculated based on the weight of rat pups, measured before the experiment (median weight 23.8 g, Supplementary Fig. 1, Supplementary Table 1). The volume of urethane solution was calculated for every animal, because of the individual and developmental variances (to increase urethane concentration for 0.5 g/kg, the median single injection volume was 0.08 ml (25% 0.07, 75% 0.083, Supplementary Fig. 1). Weight comparison between the groups of animals used for characterization of the effects of urethane and isoflurane anesthesia did not show a significant difference between the groups.

Inhalation anesthesia by isoflurane (Baxter, USA) was performed using a rodent inhalant anesthesia apparatus (E-Z Anethesia, USA). The flow rate of isoflurane was set as 0.6–0.8 l/min. The OIS acquisitions were started at a 10 min delay from the change in the anesthesia concentration. Previous studies on cortical activity in the developing mouse cortex using urethane anesthesia, showed a prominent decrease of cortical oscillatory activity observed 5–15 min after urethane injection, followed by partial recovery towards the baseline^22^. A rapid decrease of the period of activity following urethane injection was observed in different brain areas regardless of the concentration of anesthesia. The temporal sequence of the urethane quick onset and long-lasting effect of urethane was suggested to be linked to the pharmacokinetics of urethane. Isoflurane also has a short duration of action. The inductive concentration of isoflurane (5%) resulted in a significant decline of heart and respiratory rates within 10 min^79^.

### Data analysis

Statistical analysis was performed using the MATLAB Statistics toolbox. To avoid bias that may be produced by high variation in the data, comparisons between the experiments and group statistics were done using normalization of each experiment to its control value. The control values were calculated using the OIS recorded prior to the first application of the anesthetic agent. To detect the amplitude, the OIS signal was firstly convolved with a 50 point Kaiser window with shape factor 2, followed by the detection of the maximal peak value. The type of the model fit of the data was estimated based on the coefficient of determination (${\text {r}}^{2}$). The significance of developmental changes was estimated using Spearman’s rank correlation coefficient ($\rho$) with the significance level set at $p = 0.05$. The linearity of dependence was tested by estimation of the Pearson correlation coefficient with the significance level set at $p = 0.05$. Group data are expressed by box plots with the central mark indicating the median, and the bottom and top edges of the box indicating the 25th and 75th percentiles, respectively. The whiskers extend to the most extreme data points not considered outliers, and the outliers are plotted individually using the ’+’ symbol. The confidence interval (CI) was shown as a shaded area and calculated by using 2.5 Jackknife standard deviation that corresponds to a significance threshold of $p = 0.05$.

### Animal research: reporting of in vivo experiments (ARRIVE)

We state that the study was carried out in compliance with the ARRIVE guidelines. Although there is little information in the literature regarding specific signs of stress and pain in rodent pups, during our experiments we visually controlled the presence of continuous mass movements. Presence of vocalization was also used as a sign of stress of the neonatal rat pup. If these signs were present, the experimenter rechecked the positioning of the animal to eliminate the source of discomfort. If the animal continued active movements and vocalization, the experiment was stopped. However, mimicking the natural conditions for the neonatal rat maintained the animal in a stressless state in most of the experiments.

## Supplementary information

Supplementary Information.

## Data Availability

Original and processed data, signal processing and analysis routines are available on request from the corresponding author.
