# Supplementary material for: Effects of urethane and isoflurane on the sensory evoked response and local blood flow in the early postnatal rat somatosensory cortex
Source: Sci Rep. 2021 May 5;11:9567. doi: 10.1038/s41598-021-88461-8 (PMC8099888; doi:10.1038/s41598-021-88461-8)
Supplement: Supplementary file 1 — Supplementary Information. [file 41598_2021_88461_MOESM1_ESM.pdf]

Supplementary information

Effects of urethane and isoflurane on the sensory evoked response and local blood flow in the early postnatal rat somatosensory cortex.

Viktoria Shumkova, Violetta Sitdikova, Ildar Rechapov, Alexey Leukhin and Marat Minlebaev

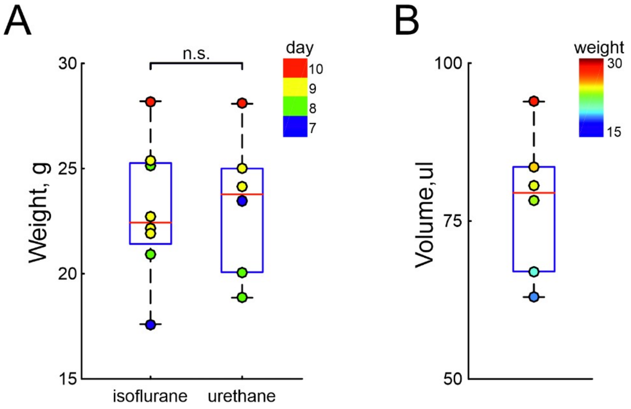

Supplementary Figure 1. Weights of the neonatal rat pups used to test the effects of isoflurane and urethane (A) and volumes of the injected 15% urethane to increase its concentration for 0.5 g/kg (B). Color map is used to code rat pups’ age on A and rat pups’ weight on B.

	median, g	25%, g	75%, g
isoflurane	22.4	21.4	25.3
urethane	23.8	20.1	25

Supplementary Table 1. Weight of the rat pups
